# Nurses and challenges faced as clinical educators: a survey of a group of nurses in Cameroon

**DOI:** 10.4314/pamj.v8i1.71085

**Published:** 2011-03-20

**Authors:** Vivian EA Eta, Mary BS Atanga, Julius Atashili, Gibson D’Cruz

**Affiliations:** 1 Faculty of Health Sciences, University of Buea, Buea, Cameroon; 2 Faculty of Health, University of East Anglia, East Anglia, United Kingdom

**Keywords:** Clinical nurse, educators-Challenges in teaching–Improvement options-Cameroon

## Abstract

**Background:**

Clinical teaching is an important component of clinical education. In nursing, clinical teaching is ensured by clinical nurse educators (CNEs). This study aimed at describing the major challenges faced by CNEs in Cameroon.

**Methods:**

In a qualitative study, supplemented with quantitative methods, CNEs were enrolled from three health districts to represent their frequency in Cameroon’s health delivery system.

**Results:**

A total of 56 CNEs participated in the study, of whom, as many as 58.9% acknowledged always facing challenges in clinical teaching and supervision. The major challenges identified were the lack of opportunities to update knowledge and skills, students’ lack of preparedness and the CNEs not being prepared for clinical teaching. CNEs attributed these challenges in major part to the lack of incentives and poor health policies.

**Conclusion:**

CNEs in Cameroon do indeed face major challenges which are of diverse origins and could adversely affect teaching in clinical settings.

## Background

Nurses constitute an important element of the medical team. A poorly trained nurse may not only hamper the team’s effectiveness but also lead to low quality health care. Clinical teaching lies at the heart of nursing education and its importance cannot be overemphasized. This is because it is in the clinical setting where student nurses are primed for the reality of their professional roles. In other words, clinical teaching and learning helps to prepare students for the kind of work they will have to do as practicing nurses. Furthermore, real life clinical experience allows student nurses to improve their skills. Therefore, clinical practice enables student nurses to become competent practitioners.

Effective clinical teaching is critical for quality nursing practices and clinical nurse educators (CNEs) are mostly responsible for teaching student nurses in the clinical setting. Despite the importance of clinical teaching to the nursing profession, in multiple settings, Cameroon inclusive, student nurses are often taught by clinical nurse educators who usually have little or no prior formal teaching [1]. Indeed, there are no guidelines to assist clinical nurse educators on how to effectively teach and supervise student nurses. As a result, they face challenges and may not adequately teach, guide, supervise and assess student nurses during clinical placements, thus potentially reducing their effectiveness as educators.

Considering that effective clinical teaching is vital for quality nursing practices, and because of the importance clinical nurse educators are to the profession, there is a need for clinical nurse educators in particular and the health system in general to identify and address the challenges faced by clinical nurse educators. An understanding of these challenges could provide a template for the clinical nurse educators to be empowered with teaching skills and thus improve teaching outcomes. This informed the objective of this study. Specifically, we sought to describe the major challenges nurses faced as clinical educators, identify the factors responsible for these challenges, assess the impact of the challenges on the quality of clinical teaching and supervision, and describe strategies used by CNEs to overcome these challenges. The ultimate goal is to provide data that could ensure good nursing practice after training.

## Methods

A qualitative study which was exploratory, descriptive and contextual was conducted to explore and describe the clinical nurse educators’ views (opinions) regarding challenges in clinical teaching in the context in which they teach and supervise students. This was supplemented by quantitative analyses. The study population was made up of clinical nurse educators who had taught for at least three years and gave their consent to participate in the study. We considered any nurse who had a role in clinical teaching or demonstration as a “clinical educator”. Eligible participants were recruited from the Ekondo Titi, Buea and Limbe Health Districts in the Ndian and Fako Division of the South West Region of Cameroon. These sites represented rural, semi-urban and urban areas respectively.

A sample of 24 clinical nurse educators were enlisted in each health district giving a total of 72 participants envisaged for the study. In each district the 24 CNEs were selected by consecutive sampling at the major hospital in the district. Questionnaires made up of both open and closed ended questions were used to collect data. Before administering the questionnaire was pre-tested to validate study questions. This was done by administering ten copies of the questionnaire to ten clinical nurse educators who were not part of the study population. Their responses confirmed the clarity and validity of the questions.

The questionnaires were then administered to the selected study participants who completed the various sections of the questionnaires. Data collected were entered into an electronic dataset.

Data were analysed using both qualitative and quantitative methods. To analyse the qualitative data, responses were read to gain understanding and insight into the clinical nurse educator’s view regarding challenges in clinical teaching. Key themes which emerged from the responses were identified and grouped into predetermined categories. For the quantitative data, frequencies of responses were determined.

This study was reviewed and authorized by the Faculty of Health Sciences, University of Buea, Cameroon. All respondents provided written consent before responding to the questionnaire.

## Results

A total of 56 out of 72 clinical nurse educators participated in the study giving a response rate of 77.8%. Approximately a-third of the participants came from each health district (Table 1). Females made up 60.4% of the participants while 39.4% were male. The age range 25-34 was the most represented (39.6%). The number of years of clinical teaching of the participants ranged from 3-25, with the range 3-5 being the most represented (40%). Sixteen percent of the participants had more than 15 years of experience. As many as 58.9% acknowledged always facing challenges in clinical teaching and supervision (Table 2). The major challenges faced by CNEs could be grouped into two: those related to their students and those related to the CNEs themselves or their environment. The most frequent challenges related to the CNEs’ students included lack of preparedness (46.4), students not taking clinical learning seriously (46.4%), dissatisfaction with duration of students’ placement (32.2%) and dissatisfaction with the number of supervisees (26.9%). Amongst the challenges related to the CNEs, the lack of opportunities to update knowledge and skills (66.1%) was the most frequently described followed by not being prepared for clinical teaching (42.9%) and the lack of working materials (17.9%).

A number of factors were enumerated as being responsible for the challenges (Table 3). These ranged from lack of financial incentives (33.9%), through poor health policies (21.5%), laxity/egoism on the part the hospital administration (12.5%) and the lack of opportunities for further learning. The lack of financial resources and high workloads were also mentioned as being responsible for challenges.

As hypothesised, the challenges had a series of negative impacts on the training of student nurses (Table 4). Study participants reported that these challenges influenced the quality of clinical teaching in many ways including making teaching difficult (21.4%), impairing effective teaching and learning (16.1%), resulting in poor teaching outcomes (12.1%), students’ objectives not being met (10.7%) and students not being taught the ideal techniques (10.7%).

A variety of strategies were reportedly employed to deal with these challenges (Table 5). Study participants reported improvising (26.8%), having extra reading (12.5%), and conducting research (10.7%) amongst others. They also described using various ways of keeping abreast with changes in nursing as a means of addressing these challenges: they accomplished this by “self-research on the internet”(26.8%), “reading books” (23.2%) and “reading nursing journals” (17.9%), amongst others.

## Discussion

There is little or no information on the challenges nurses faced as clinical educators in Cameroon. This is perhaps because of little interest on clinical teaching methods in our setting. This study which was aimed at investigating the challenges nurses faced as clinical educators in Ekondo Titi, Buea and Limbe health districts in the South West Region of Cameroon, serves as a first step in understanding the challenges this group of nurses face during clinical teaching and supervision. This study was keen on understanding these challenges because, though it is widely acknowledged that the clinical setting is an important area for student learning [2] most clinical educators may not have been formally trained. Overcoming the challenges in clinical teaching and supervision will greatly improve students’ learning, create an atmosphere for continuity and enhance nursing practice.

Results of this study revealed that a majority of the clinical educators faced difficulties during clinical teaching and supervision. This agrees with Irby [3] who holds that teaching in the clinical setting is not without challenges. The challenges were of diverse natures. Some clinical educators reported that students spent less time on clinical placement, and overall, only a few of the clinical educators were satisfied with the students’ length of stay in relation to meeting students’ objectives. According to Stein and Deese [4], student nurses need more time at the bedside to help them correlate their academic knowledge with clinical experience. Hence, students should be allowed to spend adequate length of time in clinical placements.

Students’ level of preparedness for clinical teaching was assessed by nurses as being less than average. This finding is in line with a study carried out by Penman and Oliver [5] in South Australia which indicated that students on clinical placement felt they were inadequately prepared for clinical learning. It was further revealed that students lack orientation before clinical placement as well as lack basic knowledge and skills to carryout common procedures. Therefore, concerns about students’ preparedness for clinical placements need to be examined. Students could be prepared by preceding each clinical teaching session with clearly spelled-out objectives, reading materials and demonstration in the skills lab.

Some of the clinical educators were dissatisfied with the number of students they supervised (per placement), and some of them further reported large number of students during clinical placement as challenging. In a previous study by Tamiko [6] it was found that the higher the number of students the more difficulties clinical teachers faced in teaching the students. Therefore to reduce the challenges nurses face during clinical teaching, the number of supervisee per supervisor should be moderated.

It was also found that some students did not take clinical learning seriously. The clinical educators further reported that some students were irregular, lacked respect for staff, and were not willing to learn. This is in line with Cederbaum and Klusaritz [7] who state that clinical instructors may encounter difficulties with students such as personality conflicts and lack of interest on the part of the students.

While some of the challenges (as described above) were related to students, many other challenges were related inherent to the clinical educators. Clinical nurse educators reported that they lack knowledge in certain areas of nursing and they were often challenged by some students. This study agrees with McAlister et al. [8] who state that clinical educators may be worried about the theoretical basis of their own practice. The question that often comes up is whether or not they are up to date with knowledge and their ability to teach and model skills. Effective clinical educators need to be well prepared and keep updated with current trends in nursing. In addition, clinical educators need to possess effective communication and professional teaching skills.

Nurses in general, and clinical nurse educators in particular, need to keep updated with the continuously changing nursing knowledge. In a study conducted by Morag and Lorraine [9] it was found that student expected their clinical teachers to be knowledgeable and skilled in the field of nursing. Also, according to Friendly and Roos [10] one of the most important professional responsibilities of nurses is to keep up with ever changing standard of practice. Health care is always changing therefore nurses must seek continuing education. More needs to be done to continuously update the clinical educators’ knowledge and skills.

About half of the clinical educators did not receive financial incentives for clinical teaching. Lack of incentives was also listed as one of the difficulties they faced. This implies that clinical educators may not be sufficiently motivated to teach. It was interesting to discover that even without financial incentives, this group of nurses still taught the students on clinical placements. A slight majority said they taught because of their love for the profession, while just a few of them mentioned concern for the community, and their desire to improve quality of care as reasons why they taught despite the lack of incentives. Wright [11] stipulates that nurses involved in clinical education tend to be energetic individuals with an infectious enthusiasm that comes from self confidence, excitement about nursing and pleasure in teaching. Despite the nurses’ motivation, it is still necessary to provide them with financial incentives to compensate for the time they are taking off to teach.

A high proportion of the clinical educators were not well prepared for clinical teaching. This finding are similar to those of Schönwetter et al. [1] who contend that student nurses are often taught by expert clinicians who for the most part, have limited or no prior formal teaching training. Clinical nurse educators should be well prepared and trained in order for them to produce competent nurses who will carry out safe patient care.

The study also revealed that there are difficulties such as lack of orientation for clinical teaching, lack of knowledge in some areas of nursing and lack of clinical supervision. These findings suggest that those who supervise students may be insufficiently equipped for the role. Karuhije [12], Wong and Wong [13] Mogan and Knox [14], had stipulated that there are few or no guidelines to assist clinical teachers on how to effectively supervise students so as to accomplish the students’ outcomes.

Limited time for clinical teaching was also a problem. This is in conformity with Craddock [15] who states that in a busy setting, there may be limited time for teaching and feedback. Therefore clinical educators should be well prepared in order to be equipped with clinical teaching skills to enable them maximise clinical teaching time.

The lack of working materials on the part of the students and inadequate teaching materials were further listed by nurses as challenges faced during clinical teaching and supervision. The often old and dilapidated hospital infrastructures could worsen the situation indicating that some of the challenges faced by clinical nurse educators may stem from sectors other than the health sector - thus addressing these challenges may also require a multi-sectorial approach.

The perceived factors responsible for these challenges are poor government policies, laxity on the part of the hospital administration, negligence on the part of the government and poor health care policies. Some mentioned lack of orientation to the clinical teaching task, no opportunities for further studies and heavy workloads. Nurses may be dissatisfied with their work because of their inability to attend continuing education programs or due to heavy workloads.

The challenges were perceived to have numerous consequences on the quality of clinical teaching and supervision. They made teaching difficult, impaired effective teaching and learning and led to poor teaching outcomes. Also, students’ objectives were not met and they were not taught ideal techniques.

To overcome these challenges, some of the clinical educators improvised, while others kept abreast with current changes in nursing by carrying out self research, by internet searches and by reading books. This agrees with Friendly and Roos [10] who state that, the internet provides a range of information such as tutorials, books, classes and scholarly journals which can help nurses to keep current.

It was interesting to note that, despite the absence of a formal continuous education system with certification, a substantial proportion of CNEs carried out continuous learning and research in nursing by themselves. This implies that nurses are aware of the current changes in nursing (or that nursing is constantly evolving) and they need to keep current.

A few limitations to this study need to be recognised. The use of a non-probability sample may have reduced the representativeness of our study sample. Furthermore, only public (government-run) facilities were included in this study, reducing the generalisability to CNEs in private-run facilities. A further limitation of this study is the premise that training nurse educators would increase their output as educators. We could not identify any study in a sub-Saharan setting that evaluated the impact of training and or providing incentives on CNEs, on their output. However we speculate that because students spend a considerable amount of time with CNEs, alleviating what the CNEs consider as challenges could only help improve their teaching skills. Indeed, Spencer described learning in the clinical environment as having the advantage of being motivated by its relevance and the opportunity it gives for students to be active participants [16]. On the other hand it may be difficult to implement as it tends to be difficult to standardise. Although we identify these challenges and solutions suggested by current CNEs, it is important to note that further studies will be needed to assess the impact of different incentives and CNE training in improving students’ proficiency, short term, and their impact on patient outcomes in the long run.

## Conclusion

Based on these findings it is recommended that basic working and teaching equipment be provided to the clinical nurse educators. This will enable them to effectively teach and demonstrate skills appropriately. Students should be better prepared prior to clinical postings and the number of students per educator needs to be reduced. Also, clinical nurse educators need to be kept up-to-date in nursing practice and teaching skills in order for them to adequately coach, guide, supervise and assess student nurses on clinical placements. Furthermore, the role of the clinical nurse educators can be enhanced by educational programs aimed at improving clinical teaching skills which will improve the quality of clinical teaching. Lastly, clinical nurse educators need to be supervised (supervising the supervisors) as this could go a long way to compensate any lack of adequate training as clinical educators. Further, it is recommended that further studies be conducted in a more representative sample to better understand the challenges. If these are taken into consideration it will go a long way to reduce the challenges faced by clinical nurse educators and also, make clinical teaching easier, thereby improving the quality of the nursing practice. This will in turn improve the quality of patient care and promote safe practice.

## Conflicts of interest

The authors declare that they have no competing interests.

## Authors’ contributions

All authors contributed to the conception and design, acquisition of data, or analysis and interpretation of data as well as drafting and or revising and approving the manuscript.

## Acknowledgements

We thank all the nurses who took their time off to participate in the survey.

## Figures and Tables

**Table 1:** Summary of 56 study participant characteristics

Characteristic and level	Frequency*	Percent
**Health district**		
Ekondo Titi	16	28.6
Buea	22	39.3
Limbe	18	32.1
**Gender**		
Female	29	39.6
Male	19	60.4
**Age (years)**		
Under 25	2	4.2
25-34	19	39.6
35-44	15	31.3
45-54	12	25.0
**Nurse category**		
Nurse assistant	8	16.3
Brevete Nurse	4	8.2
State Registered Nurse	25	51.0
Higher National Diploma	1	2.0
Degree Nurse	11	22.4
**Number of years of clinical teaching**		
3-5	20	40.0
6-9	9	18.0
10-14	13	26.0
15-25	8	16.0

* Totals may not all add up to 56 because of non-responses

**Table 2:** Major challenges faced by nurses as clinical educators

Challenge	Frequency	Percentage
**Related to students**		
Less than average students’ preparedness for clinical learning	26	46.4
Students do not take clinical learning seriously	26	46.4
Dissatisfaction with duration of students’ placement	18	32.2
Dissatisfaction with number of supervisees	14	26.9
Students’ irregularity	11	19.7
Lack of respect for staff	9	16.1
Students not willing to learn	9	16.1
Lack of orientation before clinical placement	6	10.7
Students lack basic knowledge and skill	5	8.9
Late coming on the part of the students	5	8.9
Students lack basic working materials	4	7.1
**Related to Clinical Nurse Educators or their environment**		
No opportunities for updates of knowledge and skills	37	66.1
Always face difficulty in clinical teaching and supervision	33	58.9
Not being prepared for clinical teaching	24	42.9
Lack of working materials	10	17.9
Lack of clinical supervision	8	14.3
Dissatisfaction with knowledge update	6	10.7
Lack of financial incentives for clinical teachers	6	10.7
Limited time for clinical teaching	3	5.4

**Table 3:** Factors responsible for challenges clinical nurse educators face

Factors	Frequency	Percent
Lack of incentives	19	33.9
Poor health policies	12	21.5
Laxity/egoism on the part of hospital	10	17.9
administration		
Negligence on the part of government	7	12.5
No opportunity for further studies	4	7.1
Lack of orientation to the clinical teaching task	4	7.1
High work loads	3	5.4
Lack financial resources	3	5.4
Students lack theoretical knowledge on certain areas	2	3.6

**Table 4:** Consequences of challenges clinical nurse educators face on the quality of clinical teaching and supervision

Consequence	Frequency	Percent
Makes teaching difficult	12	21.4
Has impaired effective teaching and learning	9	16.1
Poor teaching outcomes	7	12.5
Students objectives are not met	6	10.7
Students are not taught the ideal techniques	6	10.7
Some nursing procedures are not carried out	4	7.1
Makes teaching ineffective	4	7.1
Teaching is frustrating	3	5.4
Explain procedures without actually demonstrating	3	5.4
Embarrassed by students	3	5.4
Negatively	3	5.4
Makes Teaching boring	2	3.6
Creates an unfriendly environment	1	1.8
Avoid teaching difficult procedures	1	1.8

**Table 5:** Strategies used by clinical nurse educators to overcome challenges

Strategy	Frequency	Percent
Improvise	15	26.8
Extra reading	7	12.5
Research	6	10.7
Discussion with colleagues	5	8.9
Develop interest for further reading	4	7.1
Counsel students	1	1.8
Punish them if necessary	1	1.8
**Keep abreast with changes in nursing by:**		
Self-research by internet searches	15	26.8
Read books	13	23.2
Read nursing journals	10	17.9
Watch television	4	7.1
Attend seminars once in a while	2	3.6
Share ideas with colleagues	2	3.6
Attend clinical meetings at times	1	1.8
Ask questions from medical doctors for clarification during ward rounds	1	1.8
